# Metaphor Diffusion in Online Health Communities: Infodemiology Study in a Stroke Online Health Community

**DOI:** 10.2196/53696

**Published:** 2024-12-17

**Authors:** Sara Khoshnaw, Pietro Panzarasa, Anna De Simoni

**Affiliations:** 1 Primary Care Unit, University of Cambridge, Cambridge, United Kingdom; 2 School of Business and Management, Queen Mary University of London, London, United Kingdom; 3 Wolfson Institute of Population Health, Queen Mary University of London, London, United Kingdom

**Keywords:** online health community, social capital, metaphor, stroke, OHC, novelty, passive analysis, stroke survivor, self-promotion, post-stroke, information diffusion

## Abstract

**Background:**

Online health communities (OHCs) enable patients to create social ties with people with similar health conditions outside their existing social networks. Harnessing mechanisms of information diffusion in OHCs has attracted attention for its ability to improve illness self-management without the use of health care resources.

**Objective:**

We aimed to analyze the novelty of a metaphor used for the first time in an OHC, assess how it can facilitate self-management of post-stroke symptoms, describe its appearance over time, and classify its diffusion mechanisms.

**Methods:**

We conducted a passive analysis of posts written by UK stroke survivors and their family members in an online stroke community between 2004 and 2011. Posts including the term “legacy of stroke” were identified. Information diffusion was classified according to self-promotion or viral spread mechanisms and diffusion depth (the number of users the information spreads out to). Linguistic analysis was performed through the British National Corpus and the Google search engine.

**Results:**

Post-stroke symptoms were referred to as “legacy of stroke.” This metaphor was novel and appeared for the first time in the OHC in the second out of a total of 3459 threads. The metaphor was written by user A, who attributed it to a stroke consultant explaining post-stroke fatigue. This user was a “superuser” (ie, a user with high posting activity) and self-promoted the metaphor throughout the years in response to posts written by other users, in 51 separate threads. In total, 7 users subsequently used the metaphor, contributing to its viral diffusion, of which 3 were superusers themselves. Superusers achieved the higher diffusion depths (maximum of 3). Of the 7 users, 3 had been part of threads where user A mentioned the metaphor, while 2 users had been part of discussion threads in unrelated conversations. In total, 2 users had not been part of threads with any of the other users, suggesting that the metaphor was acquired through prior lurking activity.

**Conclusions:**

Metaphors that are considered helpful by patients with stroke to come to terms with their symptoms can diffuse in OHCs through both self-promotion and social (or viral) spreading, with the main driver of diffusion being the superuser trait. Lurking activity (the most common behavior in OHCs) contributed to the diffusion of information. As an increasing number of patients with long-term conditions join OHCs to find others with similar health-related concerns, improving clinicians’ and researchers’ awareness of the diffusion of metaphors that facilitate self-management in health social media may be beneficial beyond the individual patient.

## Introduction

Participation of people with long-term conditions in online communities can improve illness self-management [1], produce positive health-related outcomes [2-4], facilitate shared decision-making with health care professionals [5,6], and even reduce mortality [7]. There is also evidence that self-management support interventions can reduce health service utilization [8-10]. Online health community (OHC) participation leads to direct benefits in the form of information utility and social support [11,12], with information utility also helping to shape perceptions of patient empowerment among community participants [13].

One-third of stroke survivors have difficulty with communication, and half are dependent on others for daily activities due to stroke-related disabilities [14-16]. The prevalence of fatigue after stroke has been reported to be as high as 70%, yet there is currently minimal evidence on which to base an effective management strategy [17,18]. In an online stroke community, users reported several conflicting approaches to managing fatigue by their health care providers, with some patients being told to rest and others being given no information at all. As a result, stroke survivors and caregivers would seek out and offer their own, at times metaphorical, explanations [19]. Metaphors are valuable means for patients to convey new information and facilitate understanding of their symptoms [20]. They are particularly valuable for patients with long-term conditions as they help frame illness in a more manageable and hopeful way, often empowering patients as they navigate and accept their condition in daily life. Metaphors provide a means to articulate complex emotions, challenges, and symptom experiences that are difficult to express through conventional language. For instance, the commonly used “life as a journey” metaphor among dementia patients helps them and their caregivers understand and cope with the progressive nature of the illness [21]. Established and large OHCs can leverage on network characteristics [22] to spread information.

The spread of a novel metaphor in OHCs offers the opportunity to shed light on how information diffuses in OHCs. Previous studies have investigated the role of opinion leaders (superusers) in diffusing public opinions, showing that large cascades of influence are driven not by superusers but by a critical mass of easily influenced individuals [23]. Opinion leaders, though, are critical in accelerating behavioral diffusion [24]. In Twitter (now X), with respect to network features, the level of involvement of opinion leaders in diffusing a tweet increases the tweet’s structural virality [25]. Assessing information diffusion in online communities can have important implications for disease self-management and ultimately for the usage of health care services and resources.

Pei et al [26] proposed diffusion processes are induced by 3 different spreading mechanisms: (a) social spreading (or viral spreading, which occurs following social links); (b) self-promotion, through references to earlier posts by the same author; (c) broadcast, a diffusion similar to marketing, mass media, or open social media (such as Facebook or Twitter). The latter is less applicable to closed online communities. Unlike social spreading, the self-promotion mechanism relies on the dedication of the authors to repeatedly promote their own content, increasing exposure and the probability of consequent sharing. Although viral diffusion has been intensively explored in previous literature [27], the dynamics of such coupled information-spreading processes remain largely unexplored. Further research is needed to understand how each of the mechanisms associates with user traits and the outcomes of information diffusion.

In the work by Thomas et al [19], fatigue was found to be repeatedly expressed as a “legacy of stroke,” a metaphor encapsulating survivors’ experiences of a long-lasting fatigue directly linked to the stroke. Thomas initially interpreted the metaphor as reflective of how UK clinicians explained post-stroke fatigue to patients. However, subsequent discussions conducted by the authors with primary and secondary care clinicians indicated otherwise. To confirm this, a broader search of the literature and online sources was undertaken, revealing that the metaphor was indeed novel. This finding led to this study, aimed to improve our understanding of information diffusion through OHCs. We first aimed to assess the novelty and origin within the community of the metaphor “legacy of stroke.” Once established it was novel, we examined whether over time the metaphor was picked up by other users, its mechanism of diffusion, and what was the role of superusers in this process, drawing on the classification of information diffusion by Pei et al [26].

## Methods

### Design

We conducted qualitative and infodemiology analyses of stroke survivors’ posts on the archives of a moderated UK online community, based on data collected from the former qualitative study by Thomas et al [19]. We included posts written by stroke survivors and by people posting about stroke survivors between 2004 and 2011. The community underwent restructuring and changing of the host platform in early 2012, and was subsequently closed in 2012.

### Ethical Considerations

The Stroke Association provided access to the archived forum and gave their permission for the data to be used for this research purpose. Talkstroke data were stored and accessed through the University of Cambridge Clinical School Secure Data Hosting Service with reference S0126—Stroke Needs & Exp. The present analysis did not receive approval or exemption from an institutional research board, though permission to use the data was approved by the Stroke Association. Users of the forum had previously agreed that their data would become public upon registration within the forum. De Simoni et al [11] report a detailed description of the ethics linked to the research on the Talkstroke archives.

To safeguard the identity and intellectual property of participants, this analysis uses paraphrased quotes rather than direct quotations.

### Identification of Study Participants

The analysis used the archived TalkStroke online community, a UK-based, moderated online community hosted on the Stroke Association website from 2004 to 2011. In total, the TalkStroke archive contains 22,173 posts written by 2583 unique usernames [11]. The archives were searched for posts containing the terms “legacy” and “legacies.” Synonyms, misspellings, and possible abbreviations were also searched for, eg, “heritage” and “footprint.”

Participants were identified by the usernames linked to identified posts. Patients with stroke were designated as study participants, regardless of whether they were speaking in the first person or being referred to by others in the third person. The reported participant characteristics pertain therefore exclusively to the patients with stroke and do not include data on caregivers.

The top 1 percent of users by total number of posts in the dataset were named as superusers [12]. The characteristics of the stroke survivors identified, including demographics and total number of posts, were retrieved from the dataset of a previous study [11].

### Analysis

#### Novelty of the Metaphor

To explore the novelty of the metaphor, Google searches were performed for the search terms “stroke legacy” and “legacy of stroke.” The British National Corpus (BNC) [27], a database of English language, was used to search for keywords and phrases. Synonyms, misspellings, and possible abbreviations were also searched for.

#### Diffusion of the Metaphor

To analyze the diffusion of the metaphor, the first step was assigning a number to each thread in the dataset. Posts within the dataset were listed in chronological order though they did not include the time stamp. Threads including posts with the metaphor were labeled to be recognizable among others, generating a timeline of metaphor use over time. We draw on the classification of information diffusion proposed by Pei et al [26]. to study the metaphor of diffusion, as the mechanisms they describe appear to explain our data. For each participant who used the metaphor, the timeline of their participation in the community was generated through identifying threads they took part in (including in threads where the metaphor was not used) and manually highlighting in different colors the ones when they used the metaphor. Each thread was only counted once, even when users contributed to it with more than one post. Threads of posts were analyzed rather than single posts because when users take part in a thread, they are more likely not only to read posts in that thread but also to return later to check on potential new posts being added in.

Posts including the metaphor were initially identified and coded by Thomas as part of her study on post-stroke fatigue [19]. SK subsequently searched for intra-thread interactions between the users mentioning the metaphor. This was done manually by analyzing threads that included the metaphor itself and any threads with at least 2 posts from 2 study participants, using Microsoft Excel (Microsoft Corporation). Posts were analyzed and coded by SK and ADS. Lurking activity could not be studied as the dataset did not include information about logging-in activities.

## Results

### Overview

**Figure 1.**
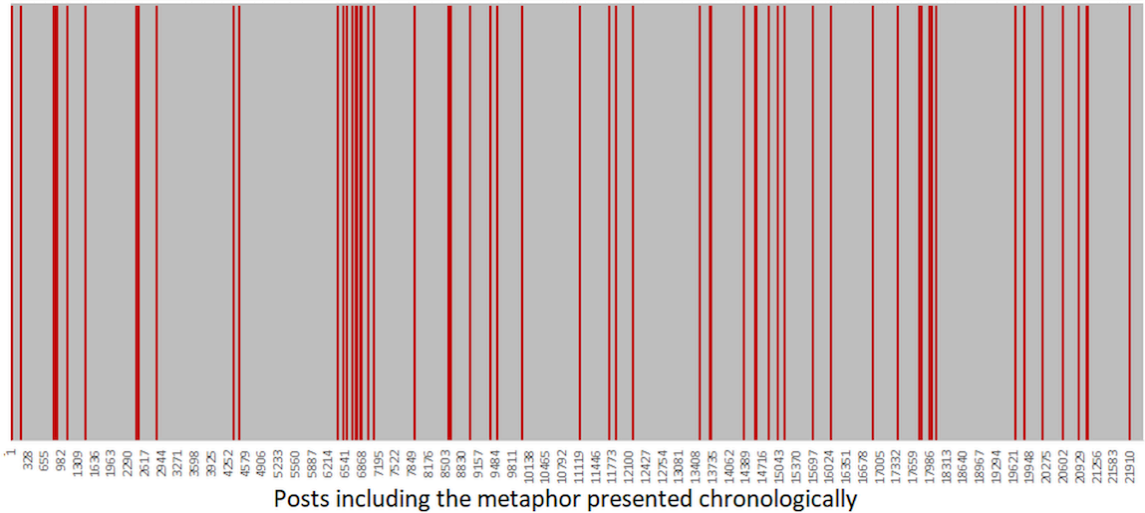
Posts including the metaphor in chronological order, among the 22,173 posts of the dataset. Posts with the metaphor are highlighted in red, while other posts are in gray.

The Talkstroke dataset included 22,173 posts in 3459 threads. 61 posts in total contained the metaphor “legacy of stroke” with the same contextual meaning (Figure 1) in separate threads. In 56% (34/61) of posts, the metaphor was used to describe fatigue, while in 44% (27/61) of posts, it referred to other post-stroke sequelae such as emotional lability, personality changes, epilepsy, depression, headache, or communication impairments.

The metaphor “fatigue is *a legacy of stroke*” appeared for the first time in the OHC in thread number 2 out of 3459, written by user A, in reply to a request for help with tiredness symptoms from a user posting about a family member who recently experienced a stroke.

User A said that fatigue is a stroke legacy and he may tire easily in future. She added that it may take long time to recover from even a small stroke.

User A attributed the metaphor to a stroke consultant explaining post-stroke fatigue in a later post.

User A wrote that after 5 years she was still getting tired. Her stroke consultant advised her that fatigue can be a major legacy of stroke. Therefore, she adapted to it: on tired days she was resting and on good days was ‘doing things.’

### Metaphor Novelty

The novelty of the construct was identified through searches within the BNC, Google and stroke OHCs. Searches of the string “fatigue is a legacy of stroke,” “fatigue is a stroke legacy,” “tiredness is a legacy of stroke,” and similar other strings did not yield results. The novelty of this metaphor lies in the term “legacy” in the context of post-stroke sequelae, used here in an unconventional manner. We took advantage of the novelty of this construct, unlikely to be created by more users at the same time, to investigate information diffusion within the OHC.

### Participants

**Figure 2.**
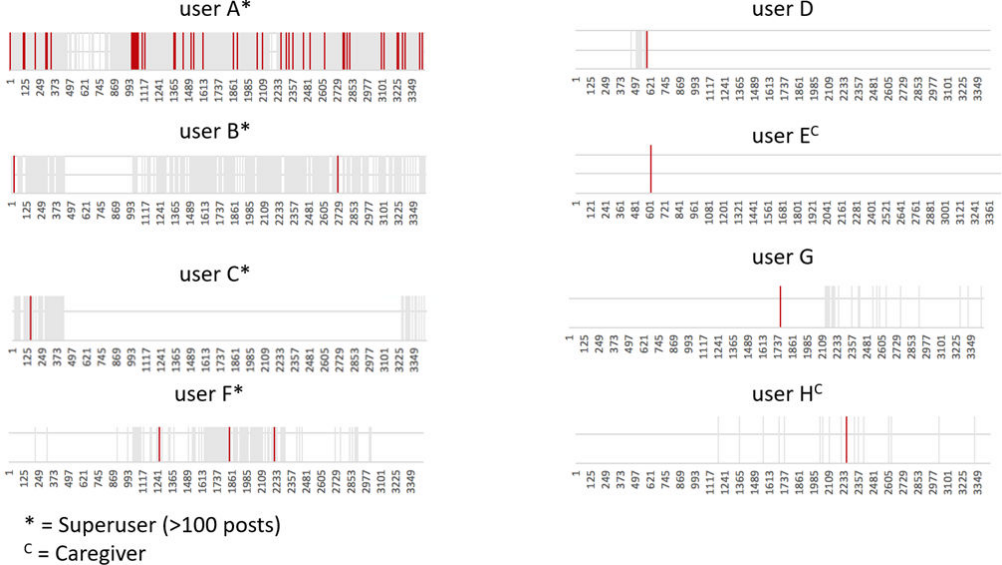
Participants’ chronological engagement in online health community threads, between 2004 and 2011. Threads including posts mentioning the metaphor are highlighted in red, while unrelated threads the users took part in are in gray.

Most posts with the metaphor were written by user A, who was a superuser (ie, a user with high posting activity, defined here as an overall contribution of >100 posts). User A self-promoted the metaphor throughout the years in reply to posts written by other users in 51 separate threads (Table 1 and Figure 2). In total, 7 other users subsequently wrote the metaphor in 10 posts in separate threads.

Of the 8 study participants, 6 were stroke survivors and 2 were people posting about family members with stroke (Table 1). The mean age of participants with stroke at the time of engagement with the community was 49 years, while the mean age at stroke was 45 years.

**Table 1.** Characteristics of participants using the metaphor.

User	Age when posting (years)	Age at stroke (years)	Time since stroke (years)	Sex	Identity	Times metaphor is used, n	Total posts in the forum, n	Superuser
A	54	46	8	F	Survivor	51	4932	Yes
B	67	55	12	M	Survivor	2	542	Yes
C	67	67	0	F	Survivor	1	178	Yes
D	55	55	0	M	Survivor	1	19	No
E	59	49	10	F	Caregiver	1	2	No
F	42	40	2	F	Survivor	3	291	Yes
G	50	47	3	M	Survivor	1	58	No
H	1	1	0	F	Caregiver	1	27	No

### Participants’ Engagement and Diffusion of the Metaphor

Participants’ engagement in threads varied, with user A being the main superuser of the community, regularly taking part in threads throughout the years, and the first to use the metaphor. Engagement of most participants was concentrated over specific time windows (ie, participants C, F, D, E, G, and H).

**Figure 3.**
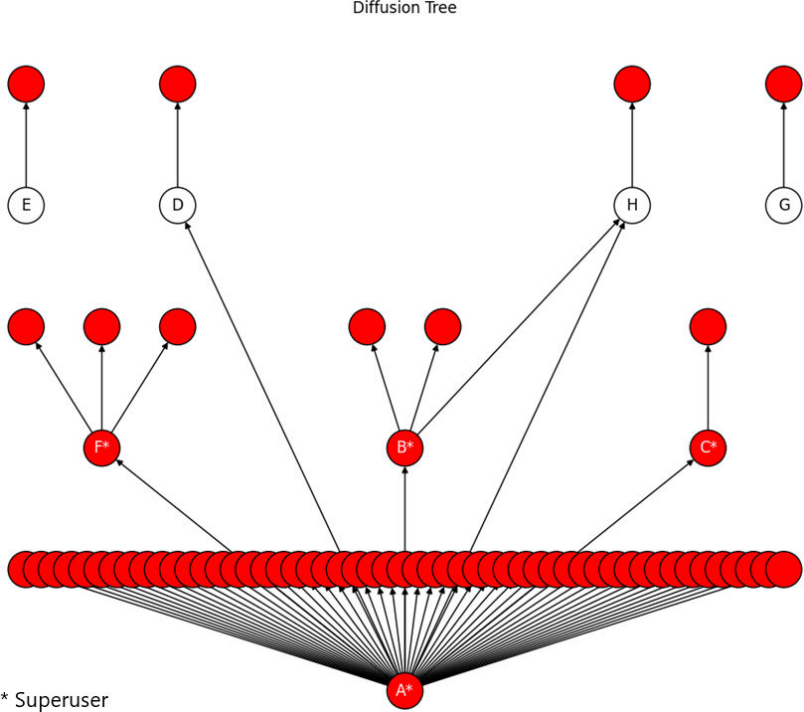
Diffusion tree within the stroke online health community. An illustration of a diffusion tree containing 68 nodes that reaches the depth of 3. Each node represents a user in the online health community, whereas each link stands for a spreading instance. In red are users who were the recipients of the metaphor from any study participants, and users B, C, and F were part of threads where the metaphor was used. Users D and H took part in unrelated threads with A and B, respectively. Users E and G were never part of threads with any of the participants.

In total, 3 superusers (B, C, and F) took part in threads where user A self-promoted the metaphor to other users. They subsequently went on to use the metaphor with other users (Figure 3). User H was part of threads unrelated to the metaphor with both users A and B. The diffusion tree contained 68 nodes, which reached a maximum depth of 3.

Users E and G were not part of any threads with other participants. We assume participants D, E, H, and G read the metaphor while lurking.

## Discussion

We found evidence supporting the diffusion of the metaphor within users of a stroke OHC, with superusers appearing to play a key role, in particular the superuser who first used and regularly self-promoted it. The metaphor and the way it was used were novel, attributed to a consultant in stroke medicine, consistently appearing throughout the 7-year OHC dataset.

Metaphors that are considered helpful by patients with stroke to come to terms with their symptoms can diffuse in OHCs through both self-promotion and social (or viral) spreading, with the main driver of diffusion being the superuser trait. Lurking activity (the most common behavior in OHCs) most likely also contributed to the diffusion of information.

A limitation of this study is that we did not analyze posts qualitatively to look for metaphors expressed by participants using different word constructs; therefore, we may have missed further diffusion. Moreover, there was no time stamp to assess the exact chronological time of the posts. Furthermore, the posts were from a relatively old dataset (2004‐2011), the number of participants identified was small, and only one metaphor was analyzed, which limit the representativeness and generalizability of the conclusions.

Searches of the metaphor using Google and the BNC were not performed using an unsupervised method, therefore limiting the claim of metaphor novelty.

Social spreading is attributed to cascades that do not exceed the depth of 3. Therefore, in online communities, most of the social spreading occurs via small and shallow information cascades. Our observation is in accordance with previous findings in other online communities [22,26,28-31].

Research has shown that tie generation in social networks can be driven by shared interests, ie, homophily breeds connections [32]. A study looking at social influence in Twitter found that highly central users who maintain social ties with a main interest group would receive retweets mainly from their own group. However, highly central users who position themselves between interest groups received more retweets from members of other interest groups than their own [33]. We raise this point as superuser A placed herself between bridging interest groups by replying to other users over a variety of topics. This could be an explanation for the use of her metaphor by users that had no interactions with her. Moreover, superuser A was the major connector (Table 1 and Figure 3) across participants. The social ties she created may have increased the likelihood of propagation of the metaphor besides the consistency of its use [34].

An important note about social networks is that they are dynamic and constantly changing over time. As a result, it is important to consider the diffusion of information over time as the network is forming and evolving. This would mean that the strength of a node or user at one time could be different at another time point. For example, at the beginning of the social network when there are very few nodes, 1 node or user who is posting little could have very high centrality, which then diminishes. Conversely, nodes can increase their centrality as time goes on, and their contribution to social influence and information spreading change correspondingly.

This study provides evidence information to improve self-management and awareness of post-stroke symptoms, diffuse in an OHC, and for the key role played by highly active users [35] in distributing its linked benefits to an entire community of patients.

There is a need to improve clinicians’ awareness of the diffusion of metaphors that facilitate self-management in health social media, as in clinical consultations patients who are active in OHCs could be encouraged to share helpful self-management metaphors.

Further studies are needed to assess whether metaphor diffusion is a feature of OHCs of other patient groups and whether health care professionals taking part in OHC together with patients could contribute to a wider and more effective diffusion.

As OHCs become more widespread, we need to further understand how to leverage on this process and its true impact on self-management. Future studies could investigate qualitatively metaphor diffusions in OHCs and their effects on self-management and quality of life.

Developing metaphors and allowing their diffusion by means of OHCs could represent a new form of health care interventions that enhances illness self-management. The relationship between metaphor diffusion and changes in sentiment expressed in posts could be explored using sentiment analysis techniques [36]. If a link between sentiment changes and metaphor diffusion is identified, this approach could be used to automatically detect the spread of metaphor-related information. Future research could further examine the impact of metaphor diffusion on self-management, as well as its potential associations with clinical or behavioral outcomes.

Research in this area will require a multidisciplinary approach from psychology, sociology, computer science, and applied mathematics, among other disciplines.

## Abbreviations

BNC: British National Corpus
OHC: online health community

## References

[1] Allen C, Vassilev I, Kennedy A, Rogers A (2016). Long-term condition self-management support in online communities: a meta-synthesis of qualitative papers. J Med Internet Res.

[2] Nesta (2015). Peer support: what is it and does it work? summarising evidence from more than 1000 studies, 2015. National Voices.

[3] Mo PKH, Coulson NS (2012). Developing a model for online support group use, empowering processes and psychosocial outcomes for individuals living with HIV/AIDS. Psychol Health.

[4] Pendry L, Salvatore J (2015). Individual and social benefits of online discussion forums. Comput Human Behav.

[5] Bartlett YK, Coulson NS (2011). An investigation into the empowerment effects of using online support groups and how this affects health professional/patient communication. Patient Educ Couns.

[6] Izuka NJ, Alexander MAW, Balasooriya-Smeekens C, Mant J, De Simoni A (2017). How do stroke survivors and their carers use practitioners’ advice on secondary prevention medications? Qualitative study of an online forum. Fam Pract.

[7] Hobbs WR, Burke M, Christakis NA, Fowler JH (2016). Online social integration is associated with reduced mortality risk. Proc Natl Acad Sci USA.

[8] Panagioti M, Richardson G, Small N (2014). Self-management support interventions to reduce health care utilisation without compromising outcomes: a systematic review and meta-analysis. BMC Health Serv Res.

[9] Taylor SJ, Pinnock H, Epiphaniou E (2014). A rapid synthesis of the evidence on interventions supporting self-management for people with long-term conditions: PRISMS – Practical systematic RevIew of Self-Management Support for long-term conditions. Health Serv Deliv Res.

[10] De Simoni A, Griffiths CJ, Taylor SJ (2016). Improving access to primary care: can online communities contribute?. Br J Gen Pract.

[11] De Simoni A, Shanks A, Balasooriya-Smeekens C, Mant J (2016). Stroke survivors and their families receive information and support on an individual basis from an online forum: descriptive analysis of a population of 2348 patients and qualitative study of a sample of participants. BMJ Open.

[12] Panzarasa P, Griffiths CJ, Sastry N, De Simoni A (2020). Social medical capital: how patients and caregivers can benefit from online social interactions. J Med Internet Res.

[13] Johnston AC, Worrell JL, di Gangi PM, Wasko M (2013). Online health communities: an assessment of the influence of participation on patient empowerment outcomes. Inf Technol People.

[14] Kelly-Hayes M, Beiser A, Kase CS, Scaramucci A, D’Agostino RB, Wolf PA (2003). The influence of gender and age on disability following ischemic stroke: the Framingham study. J Stroke Cerebrovasc Dis.

[15] Warlow C, Gijn J, Dennis M (2008). Stroke: Practical Management.

[16] Douiri A, Rudd AG, Wolfe CDA (2013). Prevalence of poststroke cognitive impairment: South London Stroke Register 1995-2010. Stroke.

[17] Glader EL, Stegmayr B, Asplund K (2002). Poststroke fatigue: a 2-year follow-up study of stroke patients in Sweden. Stroke.

[18] Wu S, Kutlubaev MA, Chun HYY (2015). Interventions for post-stroke fatigue. Cochrane Database Syst Rev.

[19] Thomas K, Gamlin C, De Simoni A, Mullis R, Mant J (2019). How is poststroke fatigue understood by stroke survivors and carers? A thematic analysis of an online discussion forum. BMJ Open.

[20] Tompkins P, Lawley J (2002). The mind, metaphor and health. Pos Health.

[21] Lempp H, Tang C, Heavey E (2023). The use of metaphors by service users with diverse long-term conditions: a secondary qualitative data analysis. Qual Res Med Healthc.

[22] Joglekar S, Sastry N, Coulson NS (2018). How online communities of people with long-term conditions function and evolve: network analysis of the structure and dynamics of the Asthma UK and British Lung Foundation Online Communities. J Med Internet Res.

[23] Watts DJ, Dodds PS (2007). Influentials, networks, and public opinion formation. J Consumer Res.

[24] Valente TW, Davis RL (1999). Accelerating the diffusion of innovations using opinion leaders. Ann Am Acad Pol Soc Sci.

[25] Meng J, Peng W, Tan PN, Liu W, Cheng Y, Bae A (2018). Diffusion size and structural virality: the effects of message and network features on spreading health information on twitter. Comput Human Behav.

[26] Pei S, Muchnik L, Tang S, Zheng Z, Makse HA (2015). Exploring the complex pattern of information spreading in online blog communities. PLoS One.

[27] British National Corpus. British National Corpus.

[28] Sha G (2010). Using Google as a super corpus to drive written language learning: a comparison with the British National Corpus. Comp Assisted Lang Learn.

[29] Goel S, Watts DJ, Goldstein DG The structure of online diffusion networks.

[30] Watts DJ, Dodds PS, Newman MEJ (2002). Identity and search in social networks. Science.

[31] Watts DJ, Dodds PS (2007). Influentials, networks, and public opinion formation. J Consum Res.

[32] Kossinets G, Watts DJ (2009). Origins of homophily in an evolving social network. Am J Sociol.

[33] Kossinets G, Watts DJ (2006). Empirical analysis of an evolving social network. Science.

[34] Wasko MM, Faraj S (2005). Why should I share? Examining social capital and knowledge contribution in electronic networks of practice. MIS Q.

[35] Dodds PS, Muhamad R, Watts DJ (2003). An experimental study of search in global social networks. Science.

[36] Pozzi FA, Fersini E, Messina E, Liu B (2016). Sentiment Analysis in Social Networks.

